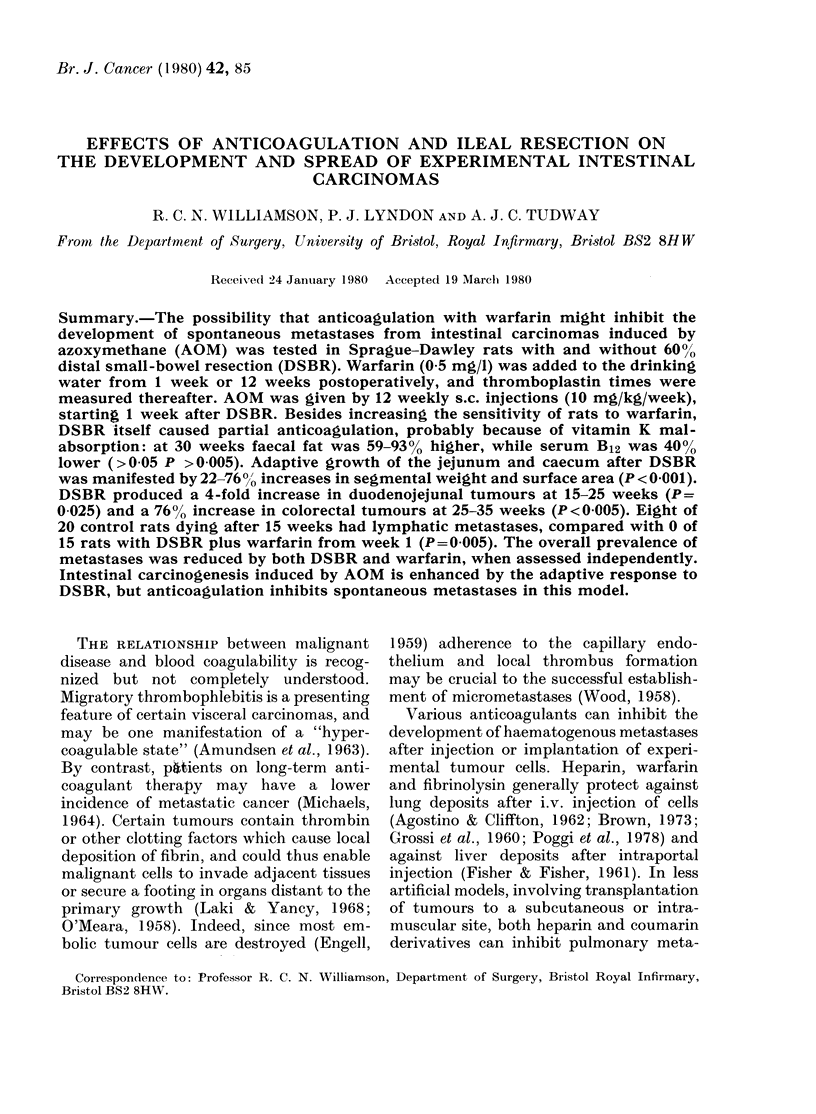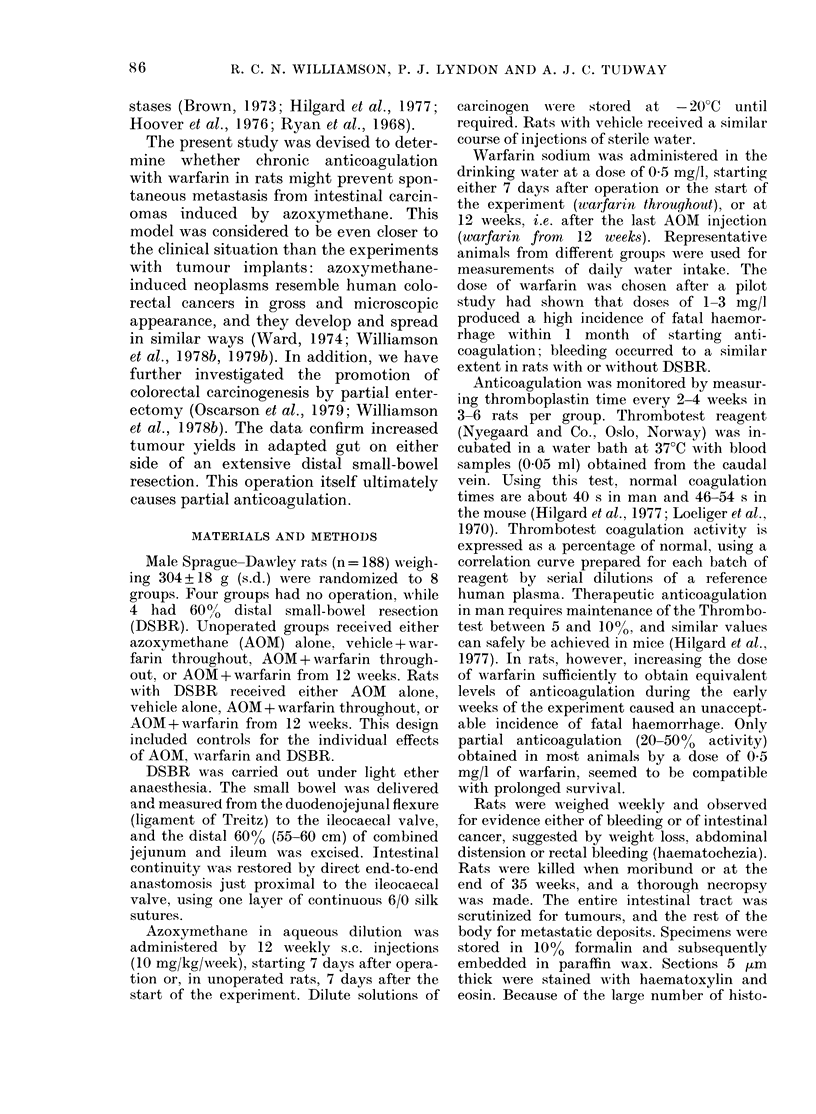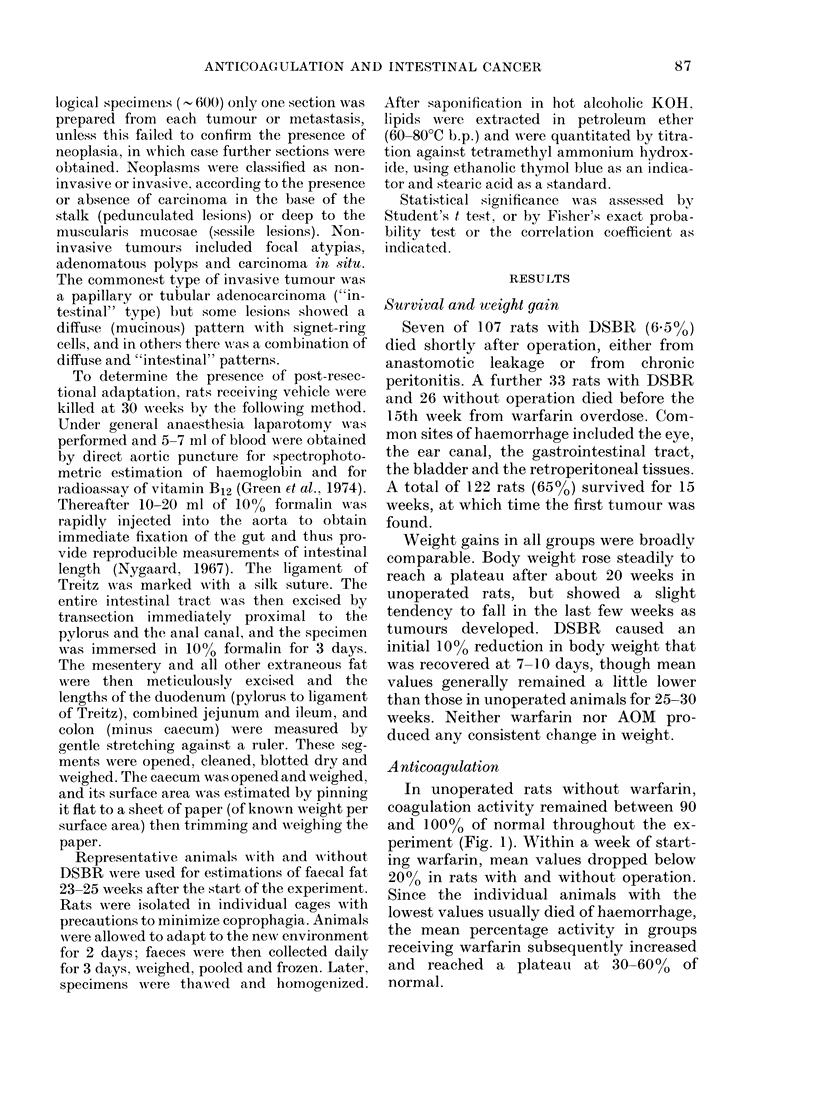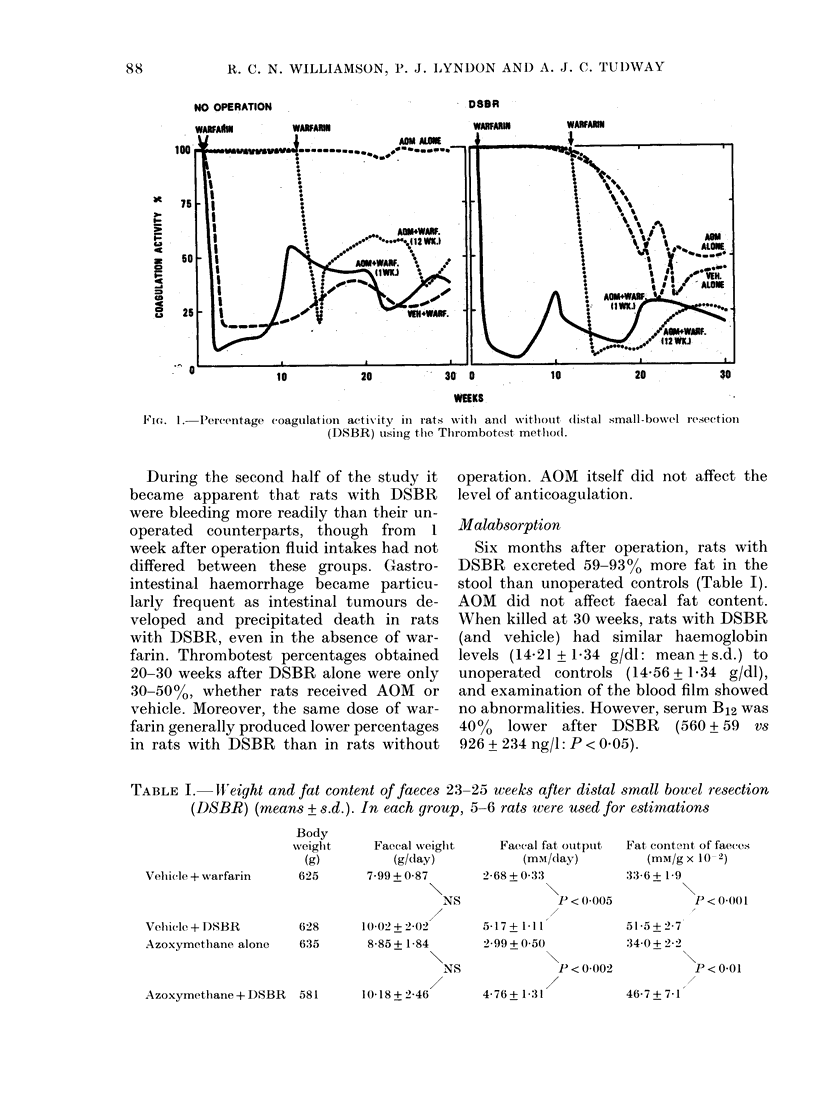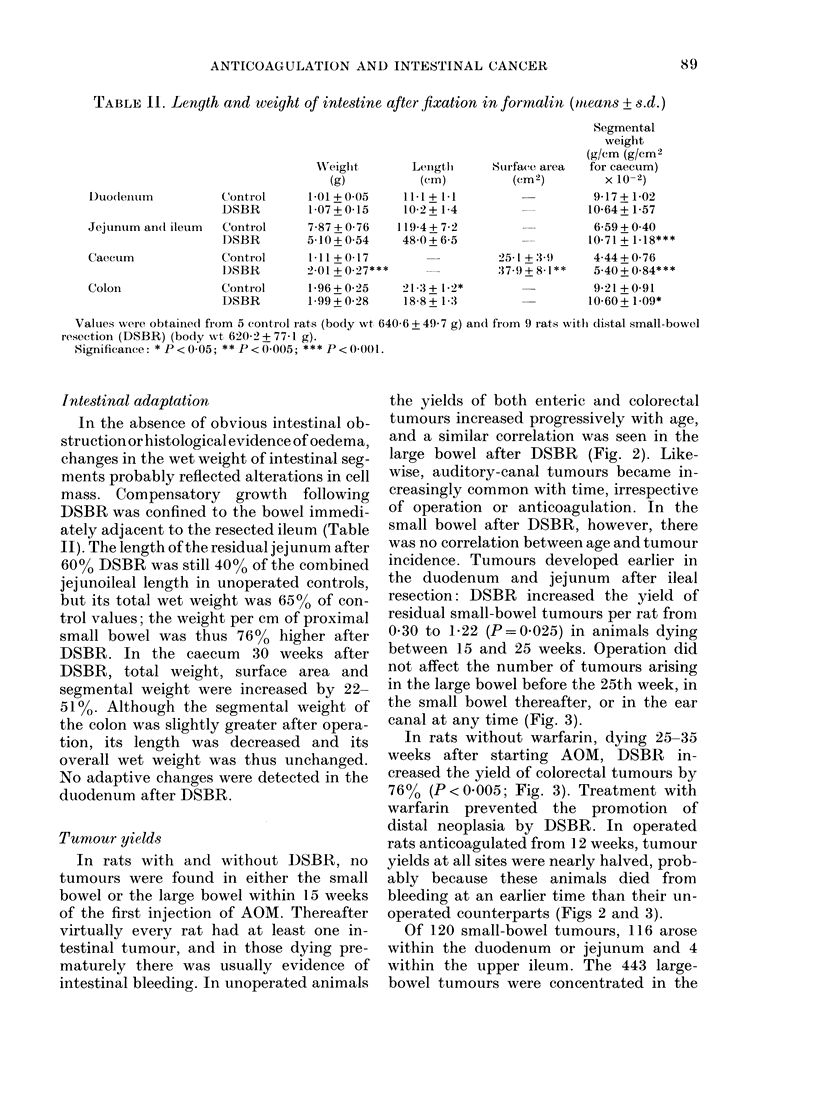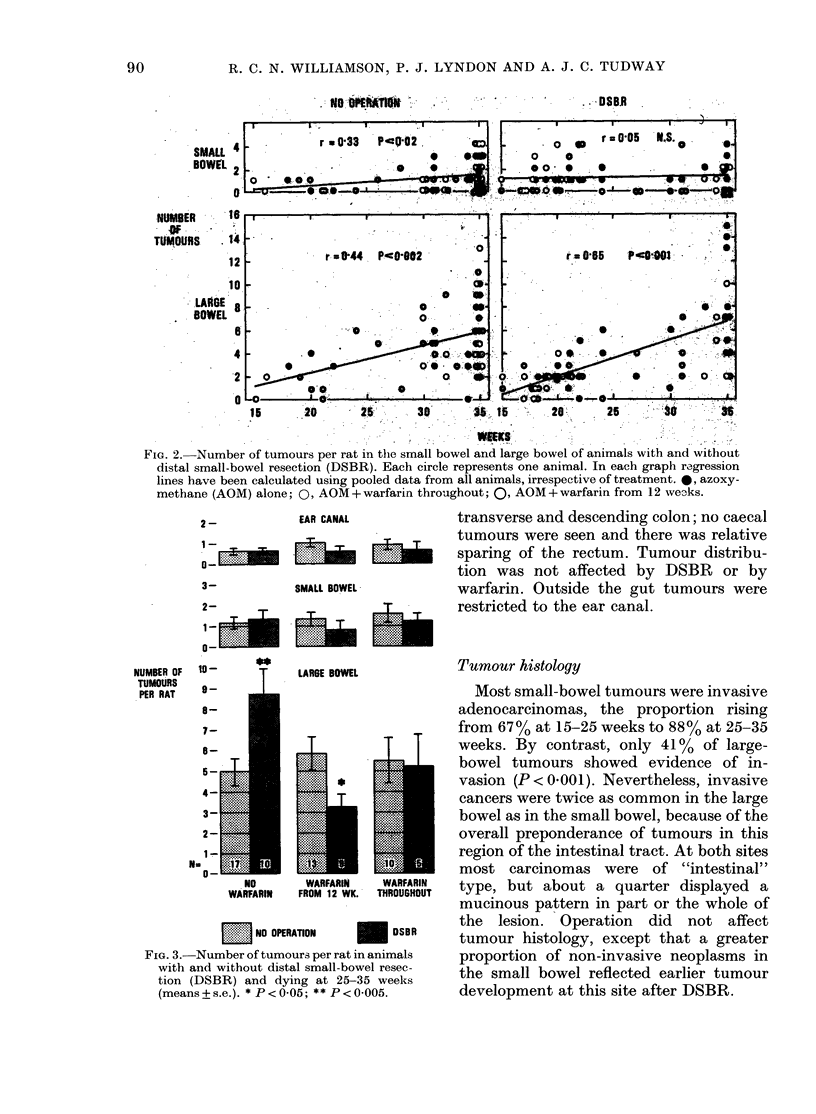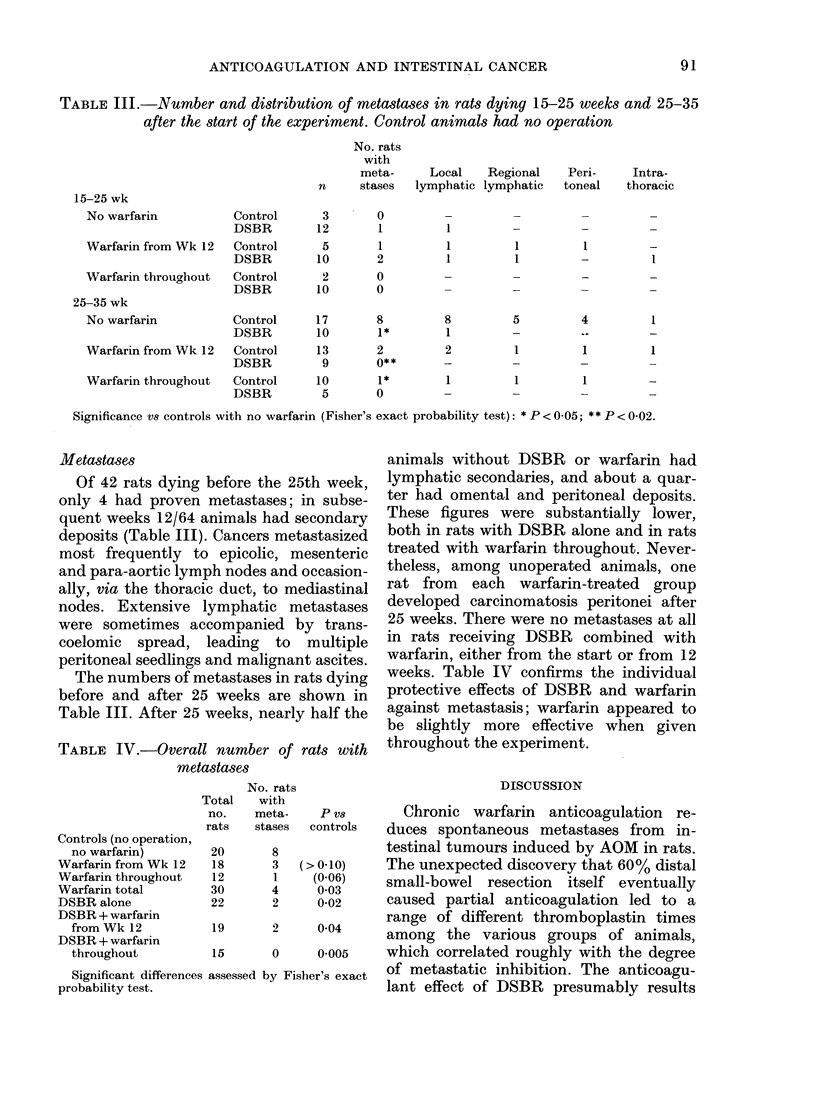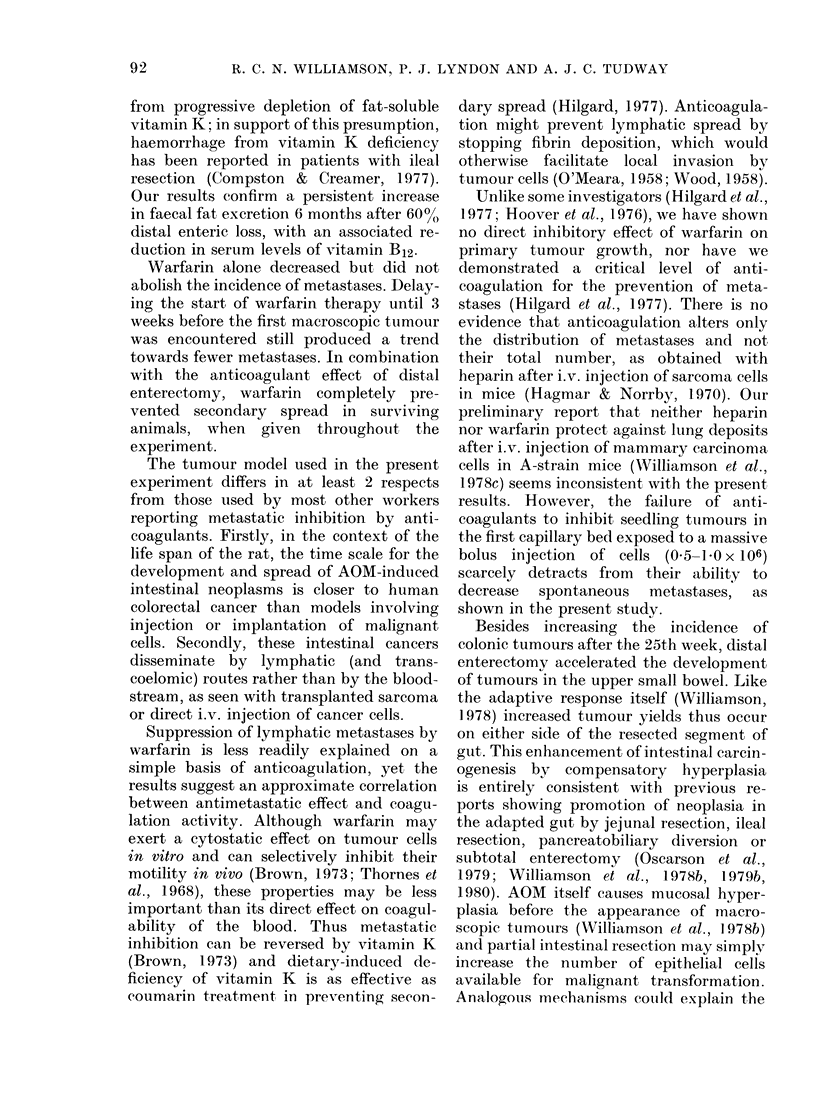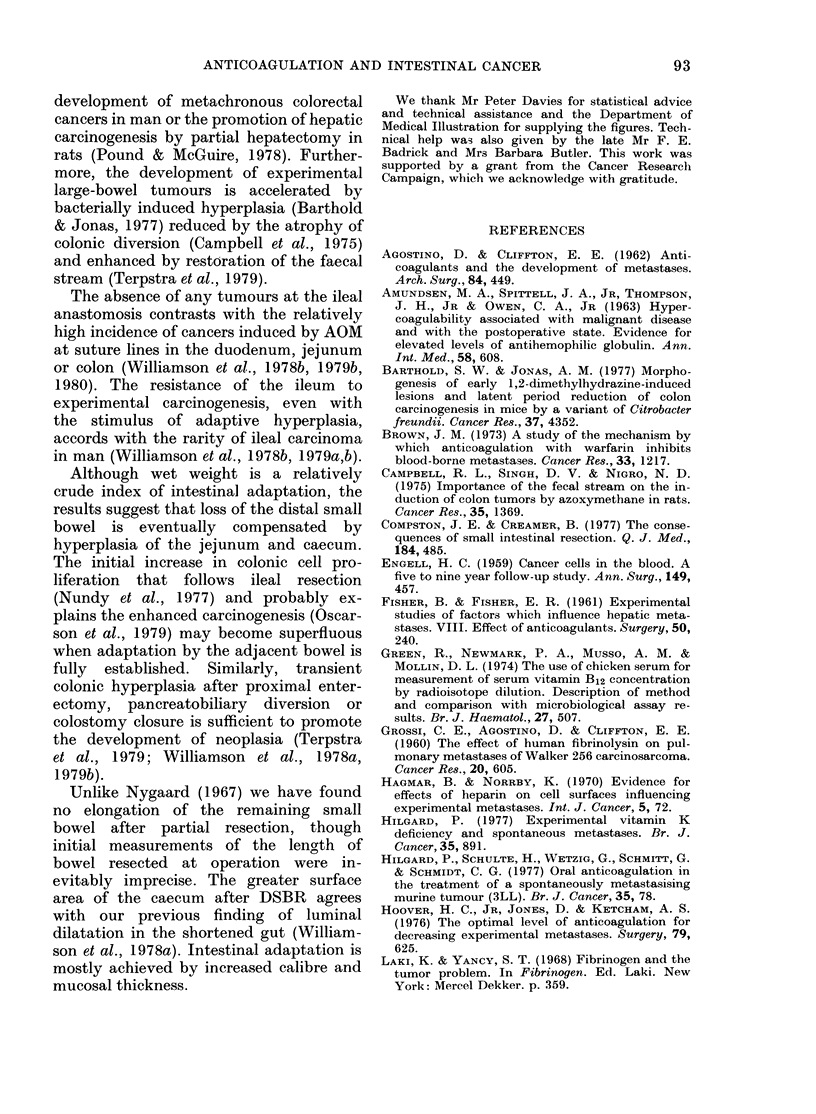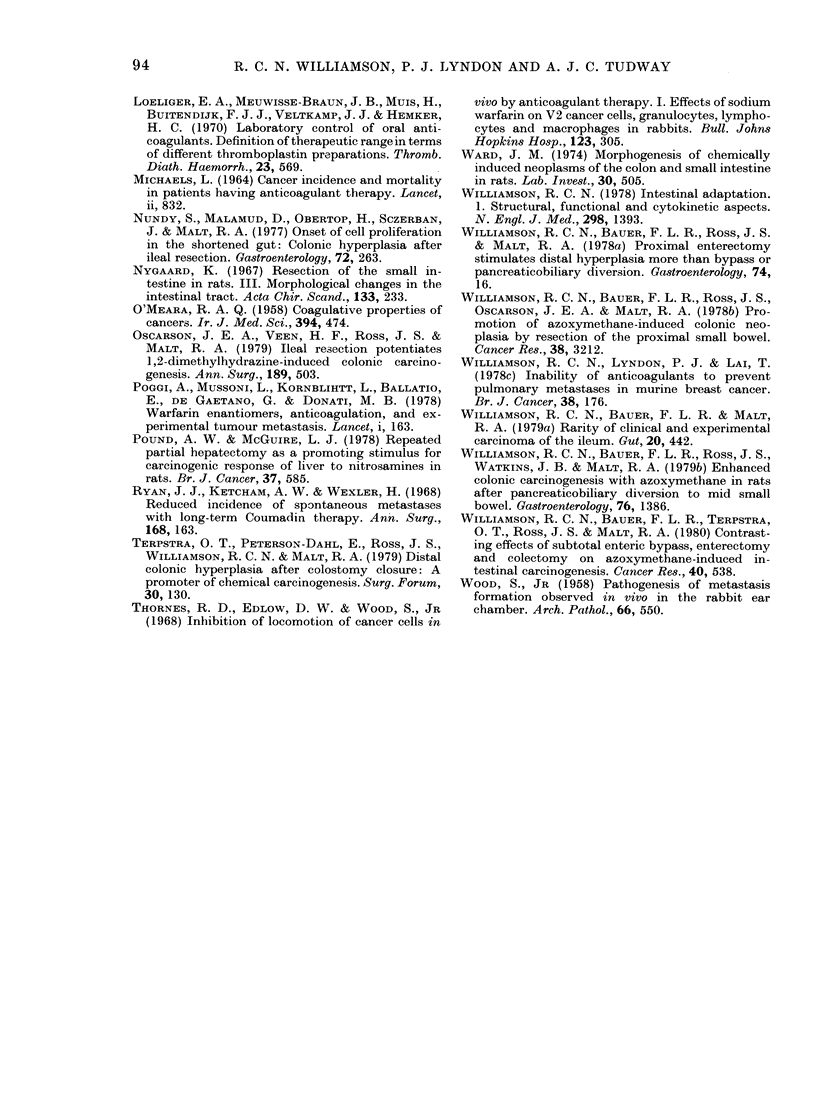# Effects of anticoagulation and ileal resection on the development and spread of experimental intestinal carcinomas.

**DOI:** 10.1038/bjc.1980.206

**Published:** 1980-07

**Authors:** R. C. Williamson, P. J. Lyndon, A. J. Tudway

## Abstract

The possibility that anticoagulation with warfarin might inhibit the development of spontaneous metastases from intestinal carcinomas induced by azoxymethane (AOM) was tested in Sprague-Dawley rats with and without 60% distal small-bowel resection (DSBR). Warfarin (0.5 mg/l) was added to the drinking water from 1 week or 12 weeks postoperatively, and thromboplastin times were measured thereafter. AOM was given by 12 weekly s.c. injections (10 mg/kg/week), starting 1 week after DSBR. Besides increasing the sensitivity of rats to warfarin, DSBR itself caused partial anticoagulation, probably because of vitamin K malabsorption: at 30 weeks faecal fat was 59-93% higher, while serum B12 was 40% lower (> 0.05 P > 0.005). Adaptive growth of the jejunum and caecum after DSBR was manifested by 22-76% increases in segmental weight and surface area (P < 0.001). DSBR produced a 4-fold increase in duodenojejunal tumours at 15-25 weeks (P = 0.025) and a 76% increase in colorectal tumours at 25-35 weeks (P < 0.005). Eight of 20 control rats dying after 15 weeks had lymphatic metastases, compared with 0 of 15 rats with DSBR plus warfarin from week 1 (P = 0.005). The overall prevalence of metastases was reduced by both DSBR and warfarin, when assessed independently. Intestinal carcinogenesis induced by AOM is enhanced by the adaptive response to DSBR, but anticoagulation inhibits spontaneous metastases in this model.


*Br. J. Cancer* (1980) **42**, 85

### EFFECTS OF ANTICOAGULATION AND ILEAL RESECTION ON THE DEVELOPMENT AND SPREAD OF EXPERIMENTAL INTESTINAL CARCINOMAS

R. C. N. WILLIAMSON, P. J. LYNDON AND A. J. C. TUDWAY

*From the Department of Surgery, University of Bristol, Royal Infirmary, Bristol BS2 8HW*

Received 24 January 1980 Accepted 19 March 1980

**Summary.—The possibility that anticoagulation with warfarin might inhibit the development of spontaneous metastases from intestinal carcinomas induced by azoxymethane (AOM) was tested in Sprague–Dawley rats with and without 60% distal small-bowel resection (DSBR). Warfarin (0·5 mg/l) was added to the drinking water from 1 week or 12 weeks postoperatively, and thromboplastin times were measured thereafter. AOM was given by 12 weekly s.c. injections (10 mg/kg/week), starting 1 week after DSBR. Besides increasing the sensitivity of rats to warfarin, DSBR itself caused partial anticoagulation, probably because of vitamin K malabsorption: at 30 weeks faecal fat was 59–93% higher, while serum $B_{12}$ was 40% lower ($>0·05\ P>0·005$). Adaptive growth of the jejunum and caecum after DSBR was manifested by 22–76% increases in segmental weight and surface area ($P<0·001$). DSBR produced a 4-fold increase in duodenojejunal tumours at 15–25 weeks ($P=0·025$) and a 76% increase in colorectal tumours at 25–35 weeks ($P<0·005$). Eight of 20 control rats dying after 15 weeks had lymphatic metastases, compared with 0 of 15 rats with DSBR plus warfarin from week 1 ($P=0·005$). The overall prevalence of metastases was reduced by both DSBR and warfarin, when assessed independently. Intestinal carcinogenesis induced by AOM is enhanced by the adaptive response to DSBR, but anticoagulation inhibits spontaneous metastases in this model.**

THE RELATIONSHIP between malignant disease and blood coagulability is recognized but not completely understood. Migratory thrombophlebitis is a presenting feature of certain visceral carcinomas, and may be one manifestation of a "hypercoagulable state" (Amundsen *et al.*, 1963). By contrast, patients on long-term anticoagulant therapy may have a lower incidence of metastatic cancer (Michaels, 1964). Certain tumours contain thrombin or other clotting factors which cause local deposition of fibrin, and could thus enable malignant cells to invade adjacent tissues or secure a footing in organs distant to the primary growth (Laki & Yancy, 1968; O'Meara, 1958). Indeed, since most embolic tumour cells are destroyed (Engell, 1959) adherence to the capillary endothelium and local thrombus formation may be crucial to the successful establishment of micrometastases (Wood, 1958).

Various anticoagulants can inhibit the development of haematogenous metastases after injection or implantation of experimental tumour cells. Heparin, warfarin and fibrinolysin generally protect against lung deposits after i.v. injection of cells (Agostino & Cliffton, 1962; Brown, 1973; Grossi *et al.*, 1960; Poggi *et al.*, 1978) and against liver deposits after intraportal injection (Fisher & Fisher, 1961). In less artificial models, involving transplantation of tumours to a subcutaneous or intramuscular site, both heparin and coumarin derivatives can inhibit pulmonary meta-

Correspondence to: Professor R. C. N. Williamson, Department of Surgery, Bristol Royal Infirmary, Bristol BS2 8HW.

stases (Brown, 1973; Hilgard *et al.*, 1977; Hoover *et al.*, 1976; Ryan *et al.*, 1968).

The present study was devised to determine whether chronic anticoagulation with warfarin in rats might prevent spontaneous metastasis from intestinal carcinomas induced by azoxymethane. This model was considered to be even closer to the clinical situation than the experiments with tumour implants: azoxymethane-induced neoplasms resemble human colorectal cancers in gross and microscopic appearance, and they develop and spread in similar ways (Ward, 1974; Williamson *et al.*, 1978*b*, 1979*b*). In addition, we have further investigated the promotion of colorectal carcinogenesis by partial enterectomy (Oscarson *et al.*, 1979; Williamson *et al.*, 1978*b*). The data confirm increased tumour yields in adapted gut on either side of an extensive distal small-bowel resection. This operation itself ultimately causes partial anticoagulation.

### MATERIALS AND METHODS

Male Sprague–Dawley rats ($n = 188$) weighing $304 \pm 18$ g (s.d.) were randomized to 8 groups. Four groups had no operation, while 4 had 60% distal small-bowel resection (DSBR). Unoperated groups received either azoxymethane (AOM) alone, vehicle + warfarin throughout, AOM + warfarin throughout, or AOM + warfarin from 12 weeks. Rats with DSBR received either AOM alone, vehicle alone, AOM + warfarin throughout, or AOM + warfarin from 12 weeks. This design included controls for the individual effects of AOM, warfarin and DSBR.

DSBR was carried out under light ether anaesthesia. The small bowel was delivered and measured from the duodenojejunal flexure (ligament of Treitz) to the ileocaecal valve, and the distal 60% (55–60 cm) of combined jejunum and ileum was excised. Intestinal continuity was restored by direct end-to-end anastomosis just proximal to the ileocaecal valve, using one layer of continuous 6/0 silk sutures.

Azoxymethane in aqueous dilution was administered by 12 weekly s.c. injections (10 mg/kg/week), starting 7 days after operation or, in unoperated rats, 7 days after the start of the experiment. Dilute solutions of carcinogen were stored at −20°C until required. Rats with vehicle received a similar course of injections of sterile water.

Warfarin sodium was administered in the drinking water at a dose of 0·5 mg/l, starting either 7 days after operation or the start of the experiment (*warfarin throughout*), or at 12 weeks, *i.e.* after the last AOM injection (*warfarin from* 12 *weeks*). Representative animals from different groups were used for measurements of daily water intake. The dose of warfarin was chosen after a pilot study had shown that doses of 1–3 mg/l produced a high incidence of fatal haemorrhage within 1 month of starting anticoagulation; bleeding occurred to a similar extent in rats with or without DSBR.

Anticoagulation was monitored by measuring thromboplastin time every 2–4 weeks in 3–6 rats per group. Thrombotest reagent (Nyegaard and Co., Oslo, Norway) was incubated in a water bath at 37°C with blood samples (0·05 ml) obtained from the caudal vein. Using this test, normal coagulation times are about 40 s in man and 46–54 s in the mouse (Hilgard *et al.*, 1977; Loeliger *et al.*, 1970). Thrombotest coagulation activity is expressed as a percentage of normal, using a correlation curve prepared for each batch of reagent by serial dilutions of a reference human plasma. Therapeutic anticoagulation in man requires maintenance of the Thrombotest between 5 and 10%, and similar values can safely be achieved in mice (Hilgard *et al.*, 1977). In rats, however, increasing the dose of warfarin sufficiently to obtain equivalent levels of anticoagulation during the early weeks of the experiment caused an unacceptable incidence of fatal haemorrhage. Only partial anticoagulation (20–50% activity) obtained in most animals by a dose of 0·5 mg/l of warfarin, seemed to be compatible with prolonged survival.

Rats were weighed weekly and observed for evidence either of bleeding or of intestinal cancer, suggested by weight loss, abdominal distension or rectal bleeding (haematochezia). Rats were killed when moribund or at the end of 35 weeks, and a thorough necropsy was made. The entire intestinal tract was scrutinized for tumours, and the rest of the body for metastatic deposits. Specimens were stored in 10% formalin and subsequently embedded in paraffin wax. Sections 5 μm thick were stained with haematoxylin and eosin. Because of the large number of histological specimens (~600) only one section was prepared from each tumour or metastasis, unless this failed to confirm the presence of neoplasia, in which case further sections were obtained. Neoplasms were classified as non-invasive or invasive, according to the presence or absence of carcinoma in the base of the stalk (pedunculated lesions) or deep to the muscularis mucosae (sessile lesions). Non-invasive tumours included focal atypias, adenomatous polyps and carcinoma *in situ*. The commonest type of invasive tumour was a papillary or tubular adenocarcinoma ("intestinal" type) but some lesions showed a diffuse (mucinous) pattern with signet-ring cells, and in others there was a combination of diffuse and "intestinal" patterns.

To determine the presence of post-resectional adaptation, rats receiving vehicle were killed at 30 weeks by the following method. Under general anaesthesia laparotomy was performed and 5–7 ml of blood were obtained by direct aortic puncture for spectrophotometric estimation of haemoglobin and for radioassay of vitamin $B_{12}$ (Green *et al.*, 1974). Thereafter 10–20 ml of 10% formalin was rapidly injected into the aorta to obtain immediate fixation of the gut and thus provide reproducible measurements of intestinal length (Nygaard, 1967). The ligament of Treitz was marked with a silk suture. The entire intestinal tract was then excised by transection immediately proximal to the pylorus and the anal canal, and the specimen was immersed in 10% formalin for 3 days. The mesentery and all other extraneous fat were then meticulously excised and the lengths of the duodenum (pylorus to ligament of Treitz), combined jejunum and ileum, and colon (minus caecum) were measured by gentle stretching against a ruler. These segments were opened, cleaned, blotted dry and weighed. The caecum was opened and weighed, and its surface area was estimated by pinning it flat to a sheet of paper (of known weight per surface area) then trimming and weighing the paper.

Representative animals with and without DSBR were used for estimations of faecal fat 23–25 weeks after the start of the experiment. Rats were isolated in individual cages with precautions to minimize coprophagia. Animals were allowed to adapt to the new environment for 2 days; faeces were then collected daily for 3 days, weighed, pooled and frozen. Later, specimens were thawed and homogenized. After saponification in hot alcoholic KOH, lipids were extracted in petroleum ether (60–80°C b.p.) and were quantitated by titration against tetramethyl ammonium hydroxide, using ethanolic thymol blue as an indicator and stearic acid as a standard.

Statistical significance was assessed by Student's *t* test, or by Fisher's exact probability test or the correlation coefficient as indicated.

### RESULTS

### *Survival and weight gain*

Seven of 107 rats with DSBR (6·5%) died shortly after operation, either from anastomotic leakage or from chronic peritonitis. A further 33 rats with DSBR and 26 without operation died before the 15th week from warfarin overdose. Common sites of haemorrhage included the eye, the ear canal, the gastrointestinal tract, the bladder and the retroperitoneal tissues. A total of 122 rats (65%) survived for 15 weeks, at which time the first tumour was found.

Weight gains in all groups were broadly comparable. Body weight rose steadily to reach a plateau after about 20 weeks in unoperated rats, but showed a slight tendency to fall in the last few weeks as tumours developed. DSBR caused an initial 10% reduction in body weight that was recovered at 7–10 days, though mean values generally remained a little lower than those in unoperated animals for 25–30 weeks. Neither warfarin nor AOM produced any consistent change in weight.

### *Anticoagulation*

In unoperated rats without warfarin, coagulation activity remained between 90 and 100% of normal throughout the experiment (Fig. 1). Within a week of starting warfarin, mean values dropped below 20% in rats with and without operation. Since the individual animals with the lowest values usually died of haemorrhage, the mean percentage activity in groups receiving warfarin subsequently increased and reached a plateau at 30–60% of normal.

FIG. 1.—Percentage coagulation activity in rats with and without distal small-bowel resection (DSBR) using the Thrombotest method.

During the second half of the study it became apparent that rats with DSBR were bleeding more readily than their unoperated counterparts, though from 1 week after operation fluid intakes had not differed between these groups. Gastrointestinal haemorrhage became particularly frequent as intestinal tumours developed and precipitated death in rats with DSBR, even in the absence of warfarin. Thrombotest percentages obtained 20–30 weeks after DSBR alone were only 30–50%, whether rats received AOM or vehicle. Moreover, the same dose of warfarin generally produced lower percentages in rats with DSBR than in rats without operation. AOM itself did not affect the level of anticoagulation.

*Malabsorption*

Six months after operation, rats with DSBR excreted 59–93% more fat in the stool than unoperated controls (Table I). AOM did not affect faecal fat content. When killed at 30 weeks, rats with DSBR (and vehicle) had similar haemoglobin levels ($14{\cdot}21 \pm 1{\cdot}34$ g/dl: mean ± s.d.) to unoperated controls ($14{\cdot}56 \pm 1{\cdot}34$ g/dl), and examination of the blood film showed no abnormalities. However, serum $B_{12}$ was 40% lower after DSBR ($560 \pm 59$ *vs* $926 \pm 234$ ng/l: $P < 0{\cdot}05$).

TABLE I.—*Weight and fat content of faeces 23–25 weeks after distal small bowel resection (DSBR) (means ± s.d.). In each group, 5–6 rats were used for estimations*

| | Body weight (g) | Faecal weight (g/day) | Faecal fat output (mM/day) | Fat content of faeces (mM/g × $10^{-2}$) |
|---|---|---|---|---|
| Vehicle + warfarin | 625 | 7·99 ± 0·87 | 2·68 ± 0·33 | 33·6 ± 1·9 |
| | | NS | $P < 0{\cdot}005$ | $P < 0{\cdot}001$ |
| Vehicle + DSBR | 628 | 10·02 ± 2·02 | 5·17 ± 1·11 | 51·5 ± 2·7 |
| Azoxymethane alone | 635 | 8·85 ± 1·84 | 2·99 ± 0·50 | 34·0 ± 2·2 |
| | | NS | $P < 0{\cdot}002$ | $P < 0{\cdot}01$ |
| Azoxymethane + DSBR | 581 | 10·18 ± 2·46 | 4·76 ± 1·31 | 46·7 ± 7·1 |

TABLE II. *Length and weight of intestine after fixation in formalin* (*means* ± *s.d.*)

| | | Weight (g) | Length (cm) | Surface area ($cm^2$) | Segmental weight (g/cm (g/$cm^2$ for caecum) $\times 10^{-2}$) |
|---|---|---|---|---|---|
| Duodenum | Control | 1·01 ± 0·05 | 11·1 ± 1·1 | — | 9·17 ± 1·02 |
| | DSBR | 1·07 ± 0·15 | 10·2 ± 1·4 | — | 10·64 ± 1·57 |
| Jejunum and ileum | Control | 7·87 ± 0·76 | 119·4 ± 7·2 | — | 6·59 ± 0·40 |
| | DSBR | 5·10 ± 0·54 | 48·0 ± 6·5 | — | 10·71 ± 1·18*** |
| Caecum | Control | 1·11 ± 0·17 | — | 25·1 ± 3·9 | 4·44 ± 0·76 |
| | DSBR | 2·01 ± 0·27*** | — | 37·9 ± 8·1** | 5·40 ± 0·84*** |
| Colon | Control | 1·96 ± 0·25 | 21·3 ± 1·2* | — | 9·21 ± 0·91 |
| | DSBR | 1·99 ± 0·28 | 18·8 ± 1·3 | — | 10·60 ± 1·09* |

Values were obtained from 5 control rats (body wt 640·6 ± 49·7 g) and from 9 rats with distal small-bowel resection (DSBR) (body wt 620·2 ± 77·1 g).
Significance: * $P < 0·05$; ** $P < 0·005$; *** $P < 0·001$.

*Intestinal adaptation*

In the absence of obvious intestinal obstruction or histological evidence of oedema, changes in the wet weight of intestinal segments probably reflected alterations in cell mass. Compensatory growth following DSBR was confined to the bowel immediately adjacent to the resected ileum (Table II). The length of the residual jejunum after 60% DSBR was still 40% of the combined jejunoileal length in unoperated controls, but its total wet weight was 65% of control values; the weight per cm of proximal small bowel was thus 76% higher after DSBR. In the caecum 30 weeks after DSBR, total weight, surface area and segmental weight were increased by 22–51%. Although the segmental weight of the colon was slightly greater after operation, its length was decreased and its overall wet weight was thus unchanged. No adaptive changes were detected in the duodenum after DSBR.

*Tumour yields*

In rats with and without DSBR, no tumours were found in either the small bowel or the large bowel within 15 weeks of the first injection of AOM. Thereafter virtually every rat had at least one intestinal tumour, and in those dying prematurely there was usually evidence of intestinal bleeding. In unoperated animals the yields of both enteric and colorectal tumours increased progressively with age, and a similar correlation was seen in the large bowel after DSBR (Fig. 2). Likewise, auditory-canal tumours became increasingly common with time, irrespective of operation or anticoagulation. In the small bowel after DSBR, however, there was no correlation between age and tumour incidence. Tumours developed earlier in the duodenum and jejunum after ileal resection: DSBR increased the yield of residual small-bowel tumours per rat from 0·30 to 1·22 ($P = 0·025$) in animals dying between 15 and 25 weeks. Operation did not affect the number of tumours arising in the large bowel before the 25th week, in the small bowel thereafter, or in the ear canal at any time (Fig. 3).

In rats without warfarin, dying 25–35 weeks after starting AOM, DSBR increased the yield of colorectal tumours by 76% ($P < 0·005$; Fig. 3). Treatment with warfarin prevented the promotion of distal neoplasia by DSBR. In operated rats anticoagulated from 12 weeks, tumour yields at all sites were nearly halved, probably because these animals died from bleeding at an earlier time than their unoperated counterparts (Figs 2 and 3).

Of 120 small-bowel tumours, 116 arose within the duodenum or jejunum and 4 within the upper ileum. The 443 large-bowel tumours were concentrated in the

FIG. 2.—Number of tumours per rat in the small bowel and large bowel of animals with and without distal small-bowel resection (DSBR). Each circle represents one animal. In each graph regression lines have been calculated using pooled data from all animals, irrespective of treatment. ●, azoxymethane (AOM) alone; ○, AOM + warfarin throughout; ◯, AOM + warfarin from 12 weeks.

FIG. 3.—Number of tumours per rat in animals with and without distal small-bowel resection (DSBR) and dying at 25–35 weeks (means ± s.e.). * $P < 0.05$; ** $P < 0.005$.

transverse and descending colon; no caecal tumours were seen and there was relative sparing of the rectum. Tumour distribution was not affected by DSBR or by warfarin. Outside the gut tumours were restricted to the ear canal.

### *Tumour histology*

Most small-bowel tumours were invasive adenocarcinomas, the proportion rising from 67% at 15–25 weeks to 88% at 25–35 weeks. By contrast, only 41% of large-bowel tumours showed evidence of invasion ($P < 0.001$). Nevertheless, invasive cancers were twice as common in the large bowel as in the small bowel, because of the overall preponderance of tumours in this region of the intestinal tract. At both sites most carcinomas were of "intestinal" type, but about a quarter displayed a mucinous pattern in part or the whole of the lesion. Operation did not affect tumour histology, except that a greater proportion of non-invasive neoplasms in the small bowel reflected earlier tumour development at this site after DSBR.

TABLE III.—*Number and distribution of metastases in rats dying* 15–25 *weeks and* 25–35 *after the start of the experiment. Control animals had no operation*

| | | *n* | No. rats with metastases | Local lymphatic | Regional lymphatic | Peritoneal | Intrathoracic |
|---|---|---|---|---|---|---|---|
| 15–25 wk | | | | | | | |
| No warfarin | Control | 3 | 0 | – | – | – | – |
| | DSBR | 12 | 1 | 1 | – | – | – |
| Warfarin from Wk 12 | Control | 5 | 1 | 1 | 1 | 1 | – |
| | DSBR | 10 | 2 | 1 | 1 | – | 1 |
| Warfarin throughout | Control | 2 | 0 | – | – | – | – |
| | DSBR | 10 | 0 | – | – | – | – |
| 25–35 wk | | | | | | | |
| No warfarin | Control | 17 | 8 | 8 | 5 | 4 | 1 |
| | DSBR | 10 | 1* | 1 | – | – | – |
| Warfarin from Wk 12 | Control | 13 | 2 | 2 | 1 | 1 | 1 |
| | DSBR | 9 | 0** | – | – | – | – |
| Warfarin throughout | Control | 10 | 1* | 1 | 1 | 1 | – |
| | DSBR | 5 | 0 | – | – | – | – |

Significance *vs* controls with no warfarin (Fisher's exact probability test): * $P < 0{\cdot}05$; ** $P < 0{\cdot}02$.

*Metastases*

Of 42 rats dying before the 25th week, only 4 had proven metastases; in subsequent weeks 12/64 animals had secondary deposits (Table III). Cancers metastasized most frequently to epicolic, mesenteric and para-aortic lymph nodes and occasionally, *via* the thoracic duct, to mediastinal nodes. Extensive lymphatic metastases were sometimes accompanied by transcoelomic spread, leading to multiple peritoneal seedlings and malignant ascites.

The numbers of metastases in rats dying before and after 25 weeks are shown in Table III. After 25 weeks, nearly half the animals without DSBR or warfarin had lymphatic secondaries, and about a quarter had omental and peritoneal deposits. These figures were substantially lower, both in rats with DSBR alone and in rats treated with warfarin throughout. Nevertheless, among unoperated animals, one rat from each warfarin-treated group developed carcinomatosis peritonei after 25 weeks. There were no metastases at all in rats receiving DSBR combined with warfarin, either from the start or from 12 weeks. Table IV confirms the individual protective effects of DSBR and warfarin against metastasis; warfarin appeared to be slightly more effective when given throughout the experiment.

TABLE IV.—*Overall number of rats with metastases*

| | Total no. rats | No. rats with metastases | *P vs* controls |
|---|---|---|---|
| Controls (no operation, no warfarin) | 20 | 8 | |
| Warfarin from Wk 12 | 18 | 3 | (> 0·10) |
| Warfarin throughout | 12 | 1 | (0·06) |
| Warfarin total | 30 | 4 | 0·03 |
| DSBR alone | 22 | 2 | 0·02 |
| DSBR + warfarin from Wk 12 | 19 | 2 | 0·04 |
| DSBR + warfarin throughout | 15 | 0 | 0·005 |

Significant differences assessed by Fisher's exact probability test.

### DISCUSSION

Chronic warfarin anticoagulation reduces spontaneous metastases from intestinal tumours induced by AOM in rats. The unexpected discovery that 60% distal small-bowel resection itself eventually caused partial anticoagulation led to a range of different thromboplastin times among the various groups of animals, which correlated roughly with the degree of metastatic inhibition. The anticoagulant effect of DSBR presumably results from progressive depletion of fat-soluble vitamin K; in support of this presumption, haemorrhage from vitamin K deficiency has been reported in patients with ileal resection (Compston & Creamer, 1977). Our results confirm a persistent increase in faecal fat excretion 6 months after 60% distal enteric loss, with an associated reduction in serum levels of vitamin $B_{12}$.

Warfarin alone decreased but did not abolish the incidence of metastases. Delaying the start of warfarin therapy until 3 weeks before the first macroscopic tumour was encountered still produced a trend towards fewer metastases. In combination with the anticoagulant effect of distal enterectomy, warfarin completely prevented secondary spread in surviving animals, when given throughout the experiment.

The tumour model used in the present experiment differs in at least 2 respects from those used by most other workers reporting metastatic inhibition by anticoagulants. Firstly, in the context of the life span of the rat, the time scale for the development and spread of AOM-induced intestinal neoplasms is closer to human colorectal cancer than models involving injection or implantation of malignant cells. Secondly, these intestinal cancers disseminate by lymphatic (and transcoelomic) routes rather than by the bloodstream, as seen with transplanted sarcoma or direct i.v. injection of cancer cells.

Suppression of lymphatic metastases by warfarin is less readily explained on a simple basis of anticoagulation, yet the results suggest an approximate correlation between antimetastatic effect and coagulation activity. Although warfarin may exert a cytostatic effect on tumour cells *in vitro* and can selectively inhibit their motility *in vivo* (Brown, 1973; Thornes *et al.*, 1968), these properties may be less important than its direct effect on coagulability of the blood. Thus metastatic inhibition can be reversed by vitamin K (Brown, 1973) and dietary-induced deficiency of vitamin K is as effective as coumarin treatment in preventing secondary spread (Hilgard, 1977). Anticoagulation might prevent lymphatic spread by stopping fibrin deposition, which would otherwise facilitate local invasion by tumour cells (O'Meara, 1958; Wood, 1958).

Unlike some investigators (Hilgard *et al.*, 1977; Hoover *et al.*, 1976), we have shown no direct inhibitory effect of warfarin on primary tumour growth, nor have we demonstrated a critical level of anticoagulation for the prevention of metastases (Hilgard *et al.*, 1977). There is no evidence that anticoagulation alters only the distribution of metastases and not their total number, as obtained with heparin after i.v. injection of sarcoma cells in mice (Hagmar & Norrby, 1970). Our preliminary report that neither heparin nor warfarin protect against lung deposits after i.v. injection of mammary carcinoma cells in A-strain mice (Williamson *et al.*, 1978*c*) seems inconsistent with the present results. However, the failure of anticoagulants to inhibit seedling tumours in the first capillary bed exposed to a massive bolus injection of cells ($0{\cdot}5$–$1{\cdot}0 \times 10^6$) scarcely detracts from their ability to decrease spontaneous metastases, as shown in the present study.

Besides increasing the incidence of colonic tumours after the 25th week, distal enterectomy accelerated the development of tumours in the upper small bowel. Like the adaptive response itself (Williamson, 1978) increased tumour yields thus occur on either side of the resected segment of gut. This enhancement of intestinal carcinogenesis by compensatory hyperplasia is entirely consistent with previous reports showing promotion of neoplasia in the adapted gut by jejunal resection, ileal resection, pancreatobiliary diversion or subtotal enterectomy (Oscarson *et al.*, 1979; Williamson *et al.*, 1978*b*, 1979*b*, 1980). AOM itself causes mucosal hyperplasia before the appearance of macroscopic tumours (Williamson *et al.*, 1978*b*) and partial intestinal resection may simply increase the number of epithelial cells available for malignant transformation. Analogous mechanisms could explain the development of metachronous colorectal cancers in man or the promotion of hepatic carcinogenesis by partial hepatectomy in rats (Pound & McGuire, 1978). Furthermore, the development of experimental large-bowel tumours is accelerated by bacterially induced hyperplasia (Barthold & Jonas, 1977) reduced by the atrophy of colonic diversion (Campbell *et al.*, 1975) and enhanced by restoration of the faecal stream (Terpstra *et al.*, 1979).

The absence of any tumours at the ileal anastomosis contrasts with the relatively high incidence of cancers induced by AOM at suture lines in the duodenum, jejunum or colon (Williamson *et al.*, 1978*b*, 1979*b*, 1980). The resistance of the ileum to experimental carcinogenesis, even with the stimulus of adaptive hyperplasia, accords with the rarity of ileal carcinoma in man (Williamson *et al.*, 1978*b*, 1979*a*,*b*).

Although wet weight is a relatively crude index of intestinal adaptation, the results suggest that loss of the distal small bowel is eventually compensated by hyperplasia of the jejunum and caecum. The initial increase in colonic cell proliferation that follows ileal resection (Nundy *et al.*, 1977) and probably explains the enhanced carcinogenesis (Oscarson *et al.*, 1979) may become superfluous when adaptation by the adjacent bowel is fully established. Similarly, transient colonic hyperplasia after proximal enterectomy, pancreatobiliary diversion or colostomy closure is sufficient to promote the development of neoplasia (Terpstra *et al.*, 1979; Williamson *et al.*, 1978*a*, 1979*b*).

Unlike Nygaard (1967) we have found no elongation of the remaining small bowel after partial resection, though initial measurements of the length of bowel resected at operation were inevitably imprecise. The greater surface area of the caecum after DSBR agrees with our previous finding of luminal dilatation in the shortened gut (Williamson *et al.*, 1978*a*). Intestinal adaptation is mostly achieved by increased calibre and mucosal thickness.

We thank Mr Peter Davies for statistical advice and technical assistance and the Department of Medical Illustration for supplying the figures. Technical help was also given by the late Mr F. E. Badrick and Mrs Barbara Butler. This work was supported by a grant from the Cancer Research Campaign, which we acknowledge with gratitude.

### REFERENCES

AGOSTINO, D. & CLIFFTON, E. E. (1962) Anticoagulants and the development of metastases. *Arch. Surg.*, **84**, 449.

AMUNDSEN, M. A., SPITTELL, J. A., JR, THOMPSON, J. H., JR & OWEN, C. A., JR (1963) Hypercoagulability associated with malignant disease and with the postoperative state. Evidence for elevated levels of antihemophilic globulin. *Ann. Int. Med.*, **58**, 608.

BARTHOLD, S. W. & JONAS, A. M. (1977) Morphogenesis of early 1,2-dimethylhydrazine-induced lesions and latent period reduction of colon carcinogenesis in mice by a variant of *Citrobacter freundii*. *Cancer Res.*, **37**, 4352.

BROWN, J. M. (1973) A study of the mechanism by which anticoagulation with warfarin inhibits blood-borne metastases. *Cancer Res.*, **33**, 1217.

CAMPBELL, R. L., SINGH, D. V. & NIGRO, N. D. (1975) Importance of the fecal stream on the induction of colon tumors by azoxymethane in rats. *Cancer Res.*, **35**, 1369.

COMPSTON, J. E. & CREAMER, B. (1977) The consequences of small intestinal resection. *Q. J. Med.*, **184**, 485.

ENGELL, H. C. (1959) Cancer cells in the blood. A five to nine year follow-up study. *Ann. Surg.*, **149**, 457.

FISHER, B. & FISHER, E. R. (1961) Experimental studies of factors which influence hepatic metastases. VIII. Effect of anticoagulants. *Surgery*, **50**, 240.

GREEN, R., NEWMARK, P. A., MUSSO, A. M. & MOLLIN, D. L. (1974) The use of chicken serum for measurement of serum vitamin $B_{12}$ concentration by radioisotope dilution. Description of method and comparison with microbiological assay results. *Br. J. Haematol.*, **27**, 507.

GROSSI, C. E., AGOSTINO, D. & CLIFFTON, E. E. (1960) The effect of human fibrinolysin on pulmonary metastases of Walker 256 carcinosarcoma. *Cancer Res.*, **20**, 605.

HAGMAR, B. & NORRBY, K. (1970) Evidence for effects of heparin on cell surfaces influencing experimental metastases. *Int. J. Cancer*, **5**, 72.

HILGARD, P. (1977) Experimental vitamin K deficiency and spontaneous metastases. *Br. J. Cancer*, **35**, 891.

HILGARD, P., SCHULTE, H., WETZIG, G., SCHMITT, G. & SCHMIDT, C. G. (1977) Oral anticoagulation in the treatment of a spontaneously metastasising murine tumour (3LL). *Br. J. Cancer*, **35**, 78.

HOOVER, H. C., JR, JONES, D. & KETCHAM, A. S. (1976) The optimal level of anticoagulation for decreasing experimental metastases. *Surgery*, **79**, 625.

LAKI, K. & YANCY, S. T. (1968) Fibrinogen and the tumor problem. In *Fibrinogen*. Ed. Laki. New York: Mercel Dekker. p. 359.

LOELIGER, E. A., MEUWISSE-BRAUN, J. B., MUIS, H., BUITENDIJK, F. J. J., VELTKAMP, J. J. & HEMKER, H. C. (1970) Laboratory control of oral anticoagulants. Definition of therapeutic range in terms of different thromboplastin preparations. *Thromb. Diath. Haemorrh.*, **23**, 569.

MICHAELS, L. (1964) Cancer incidence and mortality in patients having anticoagulant therapy. *Lancet*, ii, 832.

NUNDY, S., MALAMUD, D., OBERTOP, H., SCZERBAN, J. & MALT, R. A. (1977) Onset of cell proliferation in the shortened gut: Colonic hyperplasia after ileal resection. *Gastroenterology*, **72**, 263.

NYGAARD, K. (1967) Resection of the small intestine in rats. III. Morphological changes in the intestinal tract. *Acta Chir. Scand.*, **133**, 233.

O'MEARA, R. A. Q. (1958) Coagulative properties of cancers. *Ir. J. Med. Sci.*, **394**, 474.

OSCARSON, J. E. A., VEEN, H. F., ROSS, J. S. & MALT, R. A. (1979) Ileal resection potentiates 1,2-dimethylhydrazine-induced colonic carcinogenesis. *Ann. Surg.*, **189**, 503.

POGGI, A., MUSSONI, L., KORNBLIHTT, L., BALLATIO, E., DE GAETANO, G. & DONATI, M. B. (1978) Warfarin enantiomers, anticoagulation, and experimental tumour metastasis. *Lancet*, i, 163.

POUND, A. W. & MCGUIRE, L. J. (1978) Repeated partial hepatectomy as a promoting stimulus for carcinogenic response of liver to nitrosamines in rats. *Br. J. Cancer*, **37**, 585.

RYAN, J. J., KETCHAM, A. W. & WEXLER, H. (1968) Reduced incidence of spontaneous metastases with long-term Coumadin therapy. *Ann. Surg.*, **168**, 163.

TERPSTRA, O. T., PETERSON-DAHL, E., ROSS, J. S., WILLIAMSON, R. C. N. & MALT, R. A. (1979) Distal colonic hyperplasia after colostomy closure: A promoter of chemical carcinogenesis. *Surg. Forum*, **30**, 130.

THORNES, R. D., EDLOW, D. W. & WOOD, S., JR (1968) Inhibition of locomotion of cancer cells *in vivo* by anticoagulant therapy. I. Effects of sodium warfarin on V2 cancer cells, granulocytes, lymphocytes and macrophages in rabbits. *Bull. Johns Hopkins Hosp.*, **123**, 305.

WARD, J. M. (1974) Morphogenesis of chemically induced neoplasms of the colon and small intestine in rats. *Lab. Invest.*, **30**, 505.

WILLIAMSON, R. C. N. (1978) Intestinal adaptation. 1. Structural, functional and cytokinetic aspects. *N. Engl. J. Med.*, **298**, 1393.

WILLIAMSON, R. C. N., BAUER, F. L. R., ROSS, J. S. & MALT, R. A. (1978*a*) Proximal enterectomy stimulates distal hyperplasia more than bypass or pancreaticobiliary diversion. *Gastroenterology*, **74**, 16.

WILLIAMSON, R. C. N., BAUER, F. L. R., ROSS, J. S., OSCARSON, J. E. A. & MALT, R. A. (1978*b*) Promotion of azoxymethane-induced colonic neoplasia by resection of the proximal small bowel. *Cancer Res.*, **38**, 3212.

WILLIAMSON, R. C. N., LYNDON, P. J. & LAI, T. (1978*c*) Inability of anticoagulants to prevent pulmonary metastases in murine breast cancer. *Br. J. Cancer*, **38**, 176.

WILLIAMSON, R. C. N., BAUER, F. L. R. & MALT, R. A. (1979*a*) Rarity of clinical and experimental carcinoma of the ileum. *Gut*, **20**, 442.

WILLIAMSON, R. C. N., BAUER, F. L. R., ROSS, J. S., WATKINS, J. B. & MALT, R. A. (1979*b*) Enhanced colonic carcinogenesis with azoxymethane in rats after pancreaticobiliary diversion to mid small bowel. *Gastroenterology*, **76**, 1386.

WILLIAMSON, R. C. N., BAUER, F. L. R., TERPSTRA, O. T., ROSS, J. S. & MALT, R. A. (1980) Contrasting effects of subtotal enteric bypass, enterectomy and colectomy on azoxymethane-induced intestinal carcinogenesis. *Cancer Res.*, **40**, 538.

WOOD, S., JR (1958) Pathogenesis of metastasis formation observed *in vivo* in the rabbit ear chamber. *Arch. Pathol.*, **66**, 550.